# Acute Groin Pain Revealing Acquired Hemophilia A: A Case Report of Spontaneous Iliopsoas Hematoma

**DOI:** 10.7759/cureus.116204

**Published:** 2026-09-14

**Authors:** Sara Senne, Konstantinos Loukidis, Christophe Hälg, Nicole König, Yvonne Nussbaumer-Ochsner

**Affiliations:** 1 Internal Medicine, Cantonal Hospital Schaffhausen, Schaffhausen, CHE; 2 Hematology, Clinic Belair, Schaffhausen, CHE; 3 Radiology, Cantonal Hospital Schaffhausen, Schaffhausen, CHE; 4 Orthopedics and Traumatology, Cantonal Hospital Schaffhausen, Schaffhausen, CHE

**Keywords:** acquired hemophilia a, case report, emicizumab, factor viii inhibitor, groin pain, iliopsoas hematoma

## Abstract

Acquired hemophilia A (AHA) is a rare autoimmune bleeding disorder caused by the development of autoantibodies against coagulation factor VIII, resulting in potentially life-threatening hemorrhage. Because affected patients typically lack a personal or family history of bleeding disorders and often present with nonspecific symptoms, diagnosis may be delayed.

We report the case of a previously healthy 60-year-old woman who presented with acute progressive left groin pain caused by a spontaneous iliopsoas hematoma with active arterial bleeding. Initial laboratory evaluation revealed an isolated prolonged activated partial thromboplastin time (aPTT), while further coagulation studies demonstrated a factor VIII activity of 6% and a factor VIII inhibitor titer of 6.7 Bethesda Units, confirming the diagnosis of AHA. The patient was successfully treated with recombinant activated factor VII, high-dose corticosteroids, and emicizumab, resulting in rapid clinical improvement and initial remission. However, disease recurrence occurred during follow-up, requiring escalation of immunosuppressive therapy.

This case highlights the diagnostic challenges of AHA when presenting with atypical symptoms such as acute groin pain due to spontaneous iliopsoas hematoma. It further emphasizes that even a moderately prolonged isolated aPTT in a patient with spontaneous bleeding should prompt immediate evaluation for an acquired coagulation disorder. Early recognition and prompt multidisciplinary management are essential to prevent potentially life-threatening hemorrhagic complications.

## Introduction

Acquired hemophilia A (AHA) is a rare autoimmune bleeding disorder caused by the development of autoantibodies directed against coagulation factor VIII (FVIII), resulting in impaired coagulation and potentially life-threatening hemorrhage [1,2]. Unlike congenital hemophilia, AHA predominantly affects older adults with no personal or family history of bleeding disorders, with an estimated annual incidence of approximately 1-1.5 cases per million population [2,3]. Despite its rarity, AHA is associated with substantial morbidity and mortality [1,2]; in a national UK surveillance study, bleeding was the cause of death in 9% of patients [3].

The clinical presentation of AHA differs considerably from congenital hemophilia. Patients typically present with spontaneous bleeding into the skin, muscles, soft tissues, or mucous membranes, whereas hemarthrosis is uncommon [1,2]. Because these manifestations are often nonspecific, the diagnosis may not be considered until significant bleeding has occurred or routine laboratory investigations reveal an isolated prolongation of the activated partial thromboplastin time (aPTT). In patients with spontaneous bleeding, an isolated prolonged aPTT should prompt immediate investigation for an acquired coagulation disorder, including mixing studies, factor VIII activity, and inhibitor testing [2].

Management of AHA requires two parallel therapeutic strategies: rapid control of active bleeding using effective hemostatic agents and eradication of the FVIII inhibitor with immunosuppressive therapy [2]. Recombinant activated factor VII (rFVIIa) remains an established first-line hemostatic treatment, while emicizumab has emerged as a promising adjunctive therapeutic option in selected patients, although evidence is still evolving [2,4,5,6].

We report the case of a previously healthy 60-year-old woman who presented with acute progressive groin pain caused by a spontaneous iliopsoas hematoma. The diagnosis of AHA was established after recognition of an isolated prolonged aPTT and subsequent coagulation studies demonstrating severely reduced FVIII activity and a circulating FVIII inhibitor. This case highlights the diagnostic challenges posed by atypical presentations of AHA and emphasizes the importance of early recognition, systematic interpretation of abnormal coagulation parameters, and prompt multidisciplinary management.

This case was previously presented as a poster at the 9th Spring Congress of the Swiss Society of General Internal Medicine (SGAIM) in Basel, Switzerland, on May 21-23, 2025.

## Case presentation

A previously healthy 60-year-old woman presented to the emergency department with acute progressive pain in the left groin, which had markedly worsened over the preceding 12 hours and was aggravated by movement and weight-bearing. Approximately four weeks before presentation, she had sustained a left calf muscle strain, followed by a minor left ankle sprain ten days before admission. One week before hospitalization, she noticed spontaneous bruising involving the entire left forearm without significant trauma. She denied abdominal pain, nausea, vomiting, fever, or constitutional symptoms. Her medical history was otherwise unremarkable, with no previous bleeding episodes apart from the recently developed hematomas, no family history of bleeding disorders, and no regular medication, including anticoagulants or antiplatelet agents.

On admission, the patient appeared in considerable pain but remained hemodynamically stable. Physical examination revealed marked tenderness over the left inguinal region with pain during active and passive movement of the left hip, particularly on internal rotation. No swelling or ecchymosis of the left hip was evident on admission. No clinical signs of an inguinal or femoral hernia were present. The abdomen was soft and non-tender without signs of peritonism. Neurological examination of the lower extremities was unremarkable.

Initial laboratory investigations demonstrated an isolated prolongation of the aPTT of 45 seconds (reference range: 25-33 seconds), while the international normalized ratio (INR) and fibrinogen level (3.43 g/L) were within the normal range. The hemoglobin concentration was 12.4 g/dL on admission but declined to 9.0 g/dL over the following two days, indicating ongoing hemorrhage. The isolated prolongation of the aPTT prompted further evaluation for an underlying coagulation disorder.

Ultrasonography of the left groin demonstrated a subfascial hematoma within the iliopsoas muscle without evidence of an inguinal or femoral hernia or hip joint effusion. Contrast-enhanced computed tomography (CT) of the abdomen and pelvis confirmed a large spontaneous hematoma involving the left iliopsoas muscle. During hospitalization, the patient developed extensive spontaneous ecchymosis involving the left hip and upper thigh secondary to soft tissue extravasation from the underlying hemorrhage (Figure 1).

**Figure 1:**
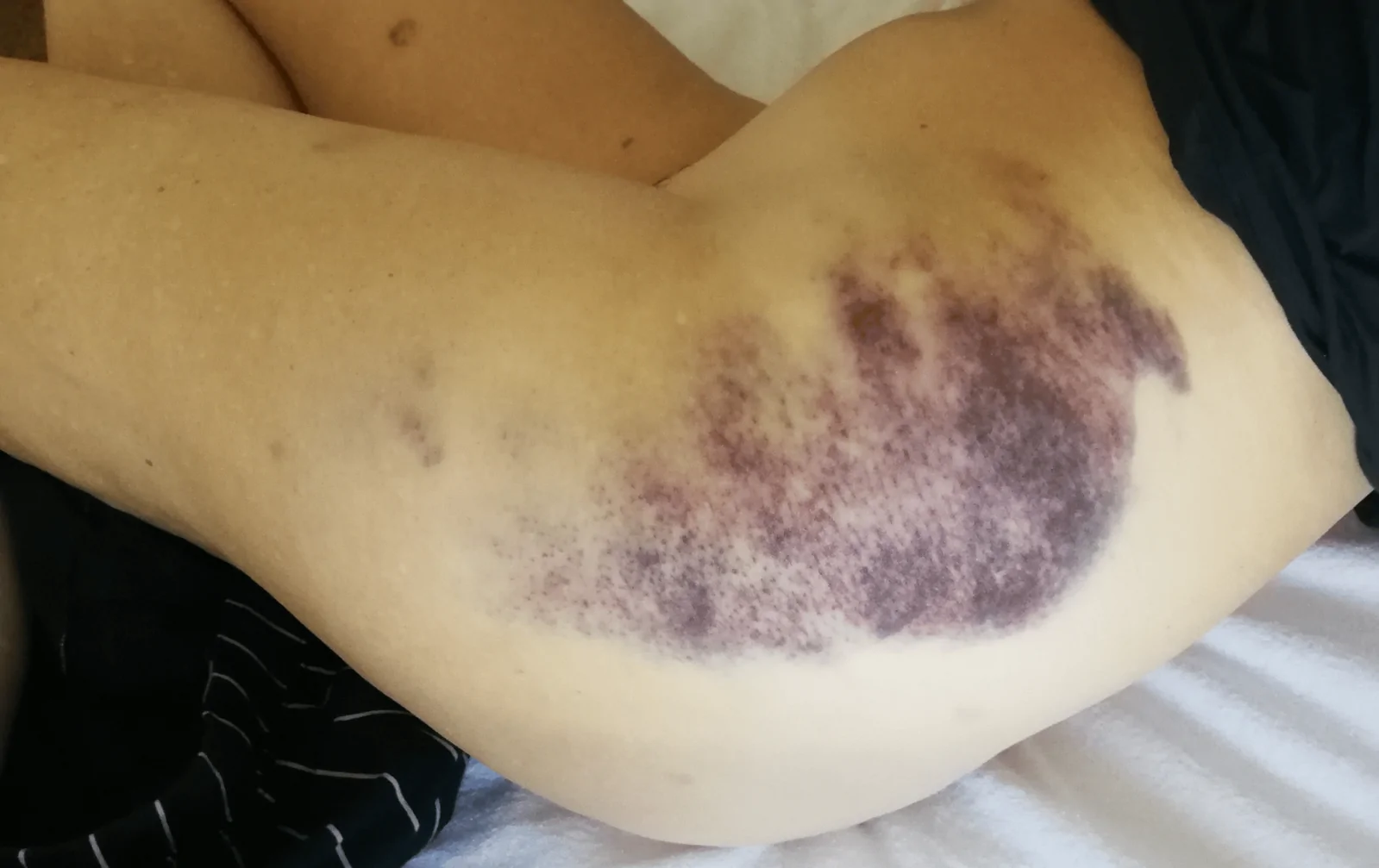
Extensive spontaneous ecchymosis involving the left hip and upper thigh in a patient with acquired hemophilia A.

CT angiography subsequently demonstrated active arterial contrast extravasation within the iliacus muscle, consistent with ongoing bleeding (Figure 2).

**Figure 2:**
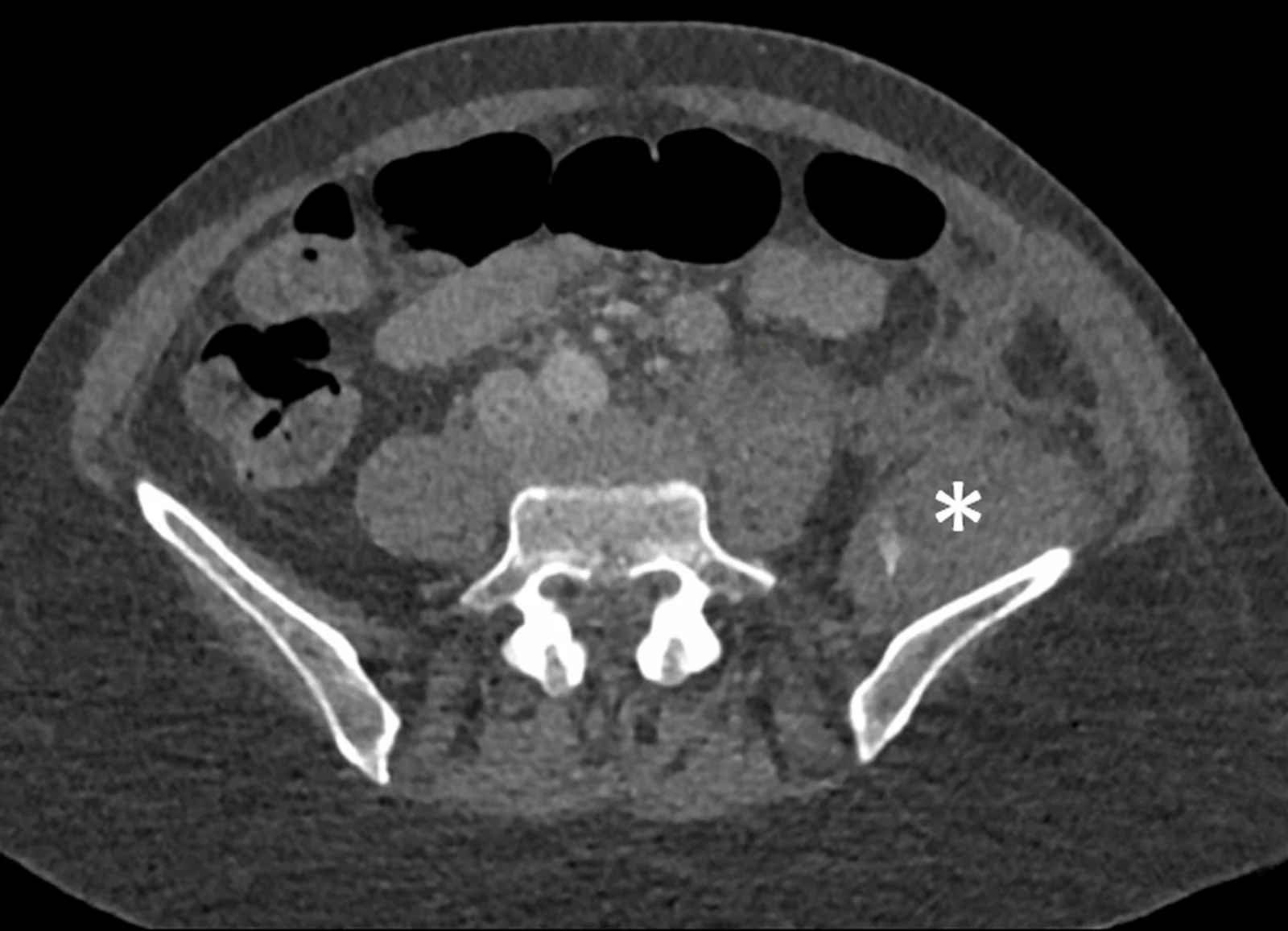
Contrast-enhanced CT angiography of the pelvis demonstrating a large left-sided iliopsoas hematoma with focal arterial contrast extravasation (asterisk), consistent with active hemorrhage.

No traumatic injury or other intra-abdominal pathology explaining the hemorrhage was identified.

Further coagulation studies revealed a factor VIII activity of 6% and a factor VIII inhibitor titer of 6.7 Bethesda Units (BU). A mixing study failed to correct the prolonged aPTT, confirming the presence of a circulating inhibitor and establishing the diagnosis of AHA. A comprehensive evaluation for underlying conditions, including autoimmune disease and occult malignancy, did not identify a secondary cause. Age-appropriate cancer screening was up to date, and the condition was therefore considered idiopathic.

Given the presence of active bleeding, hemostatic therapy with recombinant activated factor VII (rFVIIa; NovoSeven®) was initiated immediately. High-dose prednisone (100 mg daily) was started simultaneously to eradicate the factor VIII inhibitor. In addition, treatment with emicizumab (Hemlibra®), a bispecific monoclonal antibody that mimics the cofactor function of activated factor VIII, was initiated according to the recommendations of the German, Austrian, and Swiss Society on Thrombosis and Haemostasis (GTH).

The patient's clinical condition improved rapidly following initiation of therapy. Groin pain progressively resolved, allowing discontinuation of opioid analgesics, while hemoglobin levels stabilized without further evidence of bleeding. No additional hemorrhagic complications occurred, and the patient was discharged in good clinical condition with close outpatient hematology follow-up.

During follow-up, weekly emicizumab therapy was continued while corticosteroids were gradually tapered. No new bleeding manifestations occurred, and the patient initially achieved both clinical and laboratory remission, with recovery of factor VIII activity to 77% and apparent eradication of the factor VIII inhibitor. However, subsequent follow-up demonstrated disease recurrence, with factor VIII activity declining to <1% and the reappearance of the factor VIII inhibitor. In view of the relapse despite an initial response to first-line therapy, escalation of immunosuppressive treatment with rituximab was planned. The clinical course and key diagnostic findings are summarized in Table 1, while relevant laboratory findings are presented in Table 2.

**Table 1:** Chronological overview of the clinical course and key diagnostic findings. aPTT: activated partial thromboplastin time; INR: international normalized ratio; CT: computed tomography

Timeframe	Clinical events and key findings
Weeks −4 to −1 before admission	Left calf muscle strain (4 weeks before admission), minor left ankle sprain (10 days before admission), followed by spontaneous bruising of the left forearm (1 week before admission).
Day 1 (on admission)	Acute progressive left groin pain. Initial laboratory evaluation demonstrated an isolated prolonged aPTT (45 s) with normal INR and fibrinogen. Ultrasonography and contrast-enhanced CT demonstrated a large spontaneous left iliopsoas hematoma.
Day 2	Hemoglobin declined from 12.4 g/dL to 9.0 g/dL. CT angiography demonstrated active arterial contrast extravasation. Coagulation studies confirmed acquired hemophilia A (FVIII activity 6%, FVIII inhibitor titer 6.7 BU, uncorrected mixing study).
Days 2–5	Hemostatic therapy with recombinant activated factor VII (rFVIIa; NovoSeven®), high-dose prednisone (100 mg/day), and emicizumab (Hemlibra®) was initiated. Emicizumab was administered at 405 mg, followed by 210 mg on the subsequent day. Rapid clinical improvement, pain relief, and stabilization of hemoglobin levels were observed.
Discharge	Discharged in good clinical condition with ongoing weekly emicizumab therapy and gradual corticosteroid tapering.
Follow-up	Initial clinical and laboratory remission (FVIII activity 77%), followed by relapse with FVIII activity <1% and reappearance of the FVIII inhibitor. Escalation of immunosuppressive therapy with rituximab was planned.

**Table 2:** Relevant laboratory findings during diagnosis and follow-up. aPTT: activated partial thromboplastin time; INR: international normalized ratio

Time point	Parameter	Result	Reference range
Admission	Hemoglobin	12.4 g/dL	12.0–15.4 g/dL
Admission	aPTT	45 s	23–35 s
Admission	INR	1.0	<1.2
Admission	Fibrinogen	3.43 g/L	2–4 g/L
Diagnostic workup	Factor VIII activity	6%	50–200%
Diagnostic workup	Factor VIII inhibitor	6.70 BU/mL	<0.6 BU/mL
Diagnostic workup	Mixing study	Failure to correct after mixing with normal plasma	-
Initial remission	Chromogenic FVIII activity under emicizumab	77%	50–200%
Relapse	Chromogenic FVIII activity under emicizumab	<1%	50–200%
Relapse	Factor VIII inhibitor	2.3 BU/mL	<0.6 BU/mL

The combined hemostatic and immunosuppressive regimen was well tolerated. No thrombotic complications, adverse drug reactions, or catheter-related events occurred during treatment.

## Discussion

AHA remains one of the most challenging acquired bleeding disorders in clinical practice. Although rare, it is associated with considerable morbidity and mortality because patients frequently present without a personal or family history of bleeding disorders, and the initial clinical manifestations are often nonspecific [1,2]. Consequently, the diagnosis may not be considered until significant bleeding has occurred or coagulation studies reveal an isolated prolonged aPTT [2].

The present case illustrates several important diagnostic challenges. Our patient initially presented with acute groin pain, a symptom more commonly associated with musculoskeletal, orthopedic, neurological, or abdominal conditions than with an acquired coagulation disorder. Only the combination of spontaneous soft tissue bleeding and an isolated prolonged aPTT prompted further hematologic investigation, ultimately leading to the diagnosis of AHA. This case therefore emphasizes the importance of interpreting abnormal coagulation parameters within their clinical context rather than considering them incidental laboratory findings.

One of the most important clinical messages is the diagnostic significance of an isolated prolonged aPTT in patients presenting with spontaneous bleeding. Although our patient's aPTT was only moderately prolonged, she had already developed a large iliopsoas hematoma with active arterial bleeding. Previous studies have demonstrated that neither the degree of aPTT prolongation nor the residual factor VIII activity reliably predicts bleeding severity in AHA [3]. Consequently, even a modest prolongation of the aPTT should never be dismissed in patients with unexplained bleeding or spontaneous hematoma formation. Early performance of mixing studies followed by factor VIII activity and inhibitor testing remains essential for establishing the diagnosis and initiating timely treatment. Importantly, the diagnosis in our patient was triggered not by the severity of bleeding alone but by the systematic interpretation of a seemingly modest laboratory abnormality. This underscores the importance of maintaining a high index of suspicion when evaluating patients with spontaneous bleeding and an isolated prolonged aPTT.

Spontaneous iliopsoas hematoma represents an uncommon but clinically important manifestation of AHA. Patients typically present with acute groin, flank, or hip pain and may develop impaired mobility or femoral neuropathy because of compression by the expanding hematoma [1]. As these symptoms may overlap with far more common orthopedic and neurological conditions, cross-sectional imaging is crucial for confirming the diagnosis, assessing the extent of hemorrhage, and identifying active bleeding. In our patient, CT angiography demonstrated active arterial contrast extravasation, allowing prompt initiation of appropriate hemostatic therapy.

Current international recommendations advocate two parallel therapeutic strategies: rapid control of active bleeding and eradication of the factor VIII inhibitor [2]. rFVIIa remains an established first-line hemostatic agent, while immunosuppressive therapy is essential to eliminate the underlying autoantibody. More recently, emicizumab has emerged as a promising adjunctive treatment in selected patients with AHA, particularly in situations where prolonged hemostatic protection is required, although prospective evidence has only recently become available [4,6]. The substantial acquisition costs associated with both emicizumab and rFVIIa represent an important practical consideration; however, recent evidence suggests that upfront emicizumab in combination with rFVIIa may reduce overall direct treatment costs compared with rFVIIa alone [7]. In the present case, combined treatment with rFVIIa, corticosteroids, and emicizumab resulted in rapid clinical improvement, stabilization of hemoglobin levels, and initial laboratory remission.

Despite this favorable early response, our patient subsequently experienced disease recurrence with reappearance of the factor VIII inhibitor and a decline in factor VIII activity to less than 1%, necessitating escalation of immunosuppressive therapy. Although most patients achieve remission following first-line treatment, relapse has been reported and highlights the need for continued clinical and laboratory surveillance even after apparent inhibitor eradication [2,3]. Serial assessment of factor VIII activity and inhibitor titers therefore remains an essential component of long-term follow-up.

This case demonstrates that AHA should be considered in every patient presenting with spontaneous soft tissue bleeding or unexplained hematoma formation accompanied by an isolated prolonged aPTT, regardless of the degree of prolongation or the absence of a previous bleeding history. Early recognition, multidisciplinary collaboration, and prompt initiation of appropriate hemostatic and immunosuppressive therapy remain essential to reduce morbidity and improve patient outcomes.

## Conclusions

AHA should be considered in patients presenting with spontaneous soft tissue bleeding and an isolated prolonged aPTT, even in the absence of a personal or family history of bleeding disorders. This case illustrates that a seemingly modest laboratory abnormality may represent the key to diagnosing a potentially life-threatening condition. Prompt recognition, appropriate coagulation testing, and early multidisciplinary management are essential to achieve effective hemostatic control and inhibitor eradication. Although our patient initially responded well to treatment, subsequent disease recurrence highlights the importance of continued clinical and laboratory follow-up after apparent remission.
